# Use of Neuraminidase Inhibitors for Rapid Containment of Influenza: A Systematic Review and Meta-Analysis of Individual and Household Transmission Studies

**DOI:** 10.1371/journal.pone.0113633

**Published:** 2014-12-09

**Authors:** George N. Okoli, Harmony E. Otete, Charles R. Beck, Jonathan S. Nguyen-Van-Tam

**Affiliations:** Division of Epidemiology and Public Health, University of Nottingham, Nottingham, England, United Kingdom; University of Utah School of Medicine, United States of America

## Abstract

**Objectives:**

To assess the effectiveness of neuraminidase inhibitors for use in rapid containment of influenza.

**Method:**

We conducted a systematic review and meta-analysis in accordance with the PRISMA statement. Healthcare databases and sources of grey literature were searched up to 2012 and records screened against protocol eligibility criteria. Data extraction and risk of bias assessments were performed using a piloted form. Results were synthesised narratively and we undertook meta-analyses to calculate pooled estimates of effect, statistical heterogeneity and assessed publication bias.

**Findings:**

Nine randomised controlled trials (RCTs) and eight observational studies met the inclusion criteria. Neuraminidase inhibitors provided 67 to 89% protection for individuals following prophylaxis. Meta-analysis of individual protection showed a significantly lower pooled odds of laboratory confirmed seasonal or influenza A(H1N1)pdm09 infection following oseltamivir usage compared to placebo or no therapy (n = 8 studies; odds ratio (OR) = 0.11; 95% confidence interval (CI) = 0.06 to 0.20; p<0.001; I^2^ = 58.7%). This result was comparable to the pooled odds ratio for individual protection with zanamivir (OR = 0.23; 95% CI 0.16 to 0.35). Similar point estimates were obtained with widely overlapping 95% CIs for household protection with oseltamivir or zanamivir. We found no studies of neuraminidase inhibitors to prevent population-wide community transmission of influenza.

**Conclusion:**

Oseltamivir and zanamivir are effective for prophylaxis of individuals and households irrespective of treatment of the index case. There are no data which directly support an effect on wider community transmission.

**Protocol Registry:**

PROSPERO registration number: CRD42013003880

## Introduction

Influenza is a major public health concern, carrying a substantial global disease burden. Annually, an estimated 5% to 10% of adults and 20% to 30% of children are infected worldwide, with up to one million associated deaths [1]. The incubation period for influenza averages two days (range: one to four days) [2] and the mean serial interval is two to four days [3]. Consequently, influenza easily spreads rapidly through communities. Vaccination is known to be the most effective strategy for the prevention of influenza but in so many outbreak scenarios inadequacy of vaccine coverage or effectiveness, resources shortages (affordability) and urgency of the need for intervention make control with vaccine suboptimal. The high rate of antigenic drift means that vaccines must be re-formulated each year with the potential for imperfect matching between circulating influenza virus and vaccine strains [4]. Consequently, many governments stockpile antivirals, most notably, neuraminidase inhibitors, as a precaution and in preparation against influenza epidemics/pandemics. It is argued that reducing viral shedding with antiviral drugs may reduce infectivity and thereby make onward transmission of influenza less likely [5]. It has been suggested that if this phenomenon occurs in a widespread fashion, community transmission may be reduced [6].

Previous systematic reviews have demonstrated that pre- and post-exposure prophylaxis with neuraminidase inhibitors protects against laboratory confirmed influenza at individual and household levels [7]–[13] but these considered only randomised controlled trials (RCTs) of seasonal influenza conducted prior to the 2009 influenza A(H1N1) pandemic. The latest Cochrane Collaboration review on neuraminidase inhibitors for preventing and treating influenza in healthy adults and children was based on randomised, placebo controlled trials on adults and children with confirmed or suspected exposure to seasonal influenza, conducted primarily at individual and household levels [14]. Thus, the data from observational studies pertaining to transmission have not yet been summarised, and less is known about the impact of neuraminidase inhibitors for community protection against pandemic and avian influenza.

Modelling studies predicated on assumptions made from clinical studies in mainly household settings offer evidence that widespread rapid deployment of antiviral drugs around the point source of an emergent pandemic could reduce transmission and may result in containment at source [15], [16]. This concept forms the nucleus of the current World Health Organization (WHO) Rapid Containment Protocol, involving the establishment of a ‘containment zone’ [4] around the locus of emergence of a novel influenza virus, within which all asymptomatic residents will be given neuraminidase inhibitor prophylaxis for 20 days, combined with voluntary quarantine for contacts of cases, hand hygiene, social distancing and perimeter control [17]. Despite modelling simulations, it remains unclear if the findings at household level can truly be replicated at wider community level as envisaged in the Rapid Containment Protocol [18]. Furthermore, studies of pre- and post-exposure prophylaxis are often segregated when in fact under conditions of ‘rapid containment’, as envisaged by WHO, it will not be known if individuals within the containment zone are being given pre- or post-exposure prophylaxis. We therefore undertook a systematic review and meta-analysis according to the requirements of the Preferred Reporting Items for Systematic Review and Meta-Analyses (PRISMA) statement [19], deliberately combining data from pre- and post-exposure prophylaxis studies. We compared our findings to previous systematic reviews and meta-analyses and discussed the differences between our study and the previous studies.

## Methods

The systematic review protocol was registered with the National Institute for Health Research international prospective register of scientific reviews (PROSPERO) prior to executing the literature search strategy [20]. The PRISMA checklist is available as supporting information. The original study protocol was amended to clarify the review questions and eligibility criteria.

We assessed the evidence in humans that treatment of influenza cases and prophylaxis of their contacts reduce transmission. We considered all experimental and observational studies. Previous systematic reviews and meta-analyses were cross-referenced in order to identify the extent to which they had summarised all the available data and to test the sensitivity of our literature search. The study population was defined as persons of any age with laboratory confirmed influenza infection (seasonal, pandemic or avian), or with influenza-like illness (ILI), or those considered to have had close contact with any of the above persons. Laboratory confirmation was defined as a respiratory specimen which tested positive for influenza virus by reverse transcriptase-polymerase chain reaction (RT-PCR) or viral culture [21]. Symptomatic ILI was defined as an acute respiratory illness with onset during the last seven days with measured temperature ≥38°C and cough [22]. Close contact was defined as having cared for, lived with, or had direct contact with respiratory or body fluids of person or persons with laboratory confirmed influenza infection or symptomatic ILI [21]. The intervention studied was neuraminidase inhibitors (oseltamivir, zanamivir or laninamivir) whether administered as capsules, suspensions or by oral inhalation. Peramivir was not considered; being administered intravenously, it is unsuitable for use at community level. Eligible comparators included no treatment, placebo, or sham antivirals, although we also included studies which did not use a comparator. The outcome measure was community transmission which, in the absence of an internationally accepted definition of what constitutes a community setting, we defined as: epidemiologically linked cases in settings other than hospitals, care homes, nursing homes, boarding schools, and places of detention.

### Search strategy

One reviewer searched healthcare databases and other literature sources to identify published and unpublished literature on human subjects, in any language, up to December 2012 (see S1 Table: Literature search sources). The search was based on the term construct used for MEDLINE described in the review protocol, adapted for other literature sources where necessary (see S2 Table: Literature search terms). Reference and citation tracking were undertaken to identify further relevant studies. Relevant manufacturers (Roche, GlaxoSmithKline, and Biota Holdings Ltd) and domain experts were contacted for possibly relevant literature to screen for inclusion.

### Study selection

Identified articles were imported into EndNote software X4.0.2 (Thomson Reuters, California, USA) and screened by two reviewers after removal of duplicates. The protocol eligibility criteria were applied using a three stage sequential sifting approach to review title, abstract and full text [20]. Sifting was performed in parallel by two reviewers (GNO, HEO), with any disagreements discussed and resolved via involvement of a third reviewer (CRB).

### Data extraction

Data extraction was carried out, in parallel, by two reviewers (GNO, HEO) using a standardised, piloted template; a third reviewer (CRB) resolved any disagreements. The data extraction form is available as an appendix to the study protocol [20].

### Risk of bias assessment

The risk of bias in individual studies was assessed at both the study and outcome level in compliance with the PRISMA statement [19]. The risk of bias in experimental and prospective cohort studies was assessed using the Cochrane Collaboration tool [23]. The Newcastle Ottawa scale was used for assessing risk of bias in other observational studies [24].

### Result synthesis and analysis

A narrative approach was used to synthesise quality assessments according to a recognised framework [25]. Sub-analyses were planned to describe differences between: pre-exposure prophylaxis, post-exposure prophylaxis without treatment of index case, post-exposure prophylaxis with treatment of index case, and treatment of index case only; seasonal, pandemic and avian influenza; and neuraminidase inhibitor type. Meta-analysis was conducted where feasible, using a random effects model, in Stata version 12 (StatCorp LP, Texas, USA). Pooled estimates of effect were calculated using odds ratios (OR) including 95% confidence intervals (CIs). Statistical heterogeneity was assessed through calculation of I^2^. Sensitivity analyses were undertaken for RCTs of seasonal influenza and observational studies of influenza A(H1N1)pdm09. Publication bias was assessed for each outcome measure, visually using funnel plots of effect size versus sample size for each included study, and statistically using Egger’s regression test.

## Results

**Figure 1:**
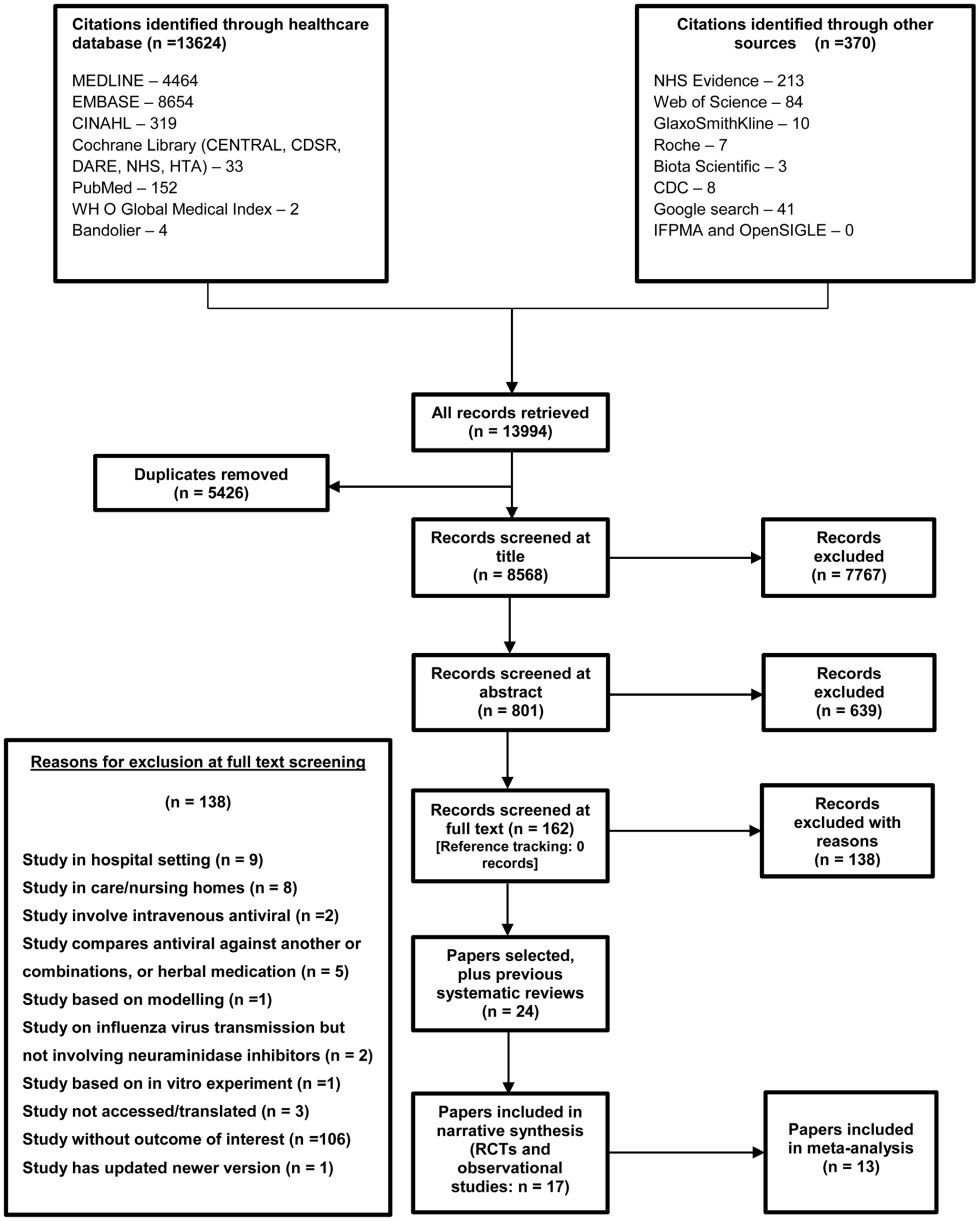
Summary of the literature search and sifting process (PRISMA flow diagram). CENTRAL = Cochrane Central Register of Controlled Trials; CDSR = Cochrane Database of Systematic Reviews; DARE = Database of Abstracts of Reviews; NHS = National Health Services; HTA = Health Technology Assessment; WHO Global Medical Index = World Health Organization Global Medical Index; OpenSIGLE = System for information on Grey Literature in Europe; CDC = Centers for Diseases Control and Prevention; IFPMA = International Federation of Pharmaceutical Manufacturers Associations.

A total of 13,994 records were identified from all sources. After removing duplicates, 8,568 remained; sifting revealed 17 eligible studies (summarised in Fig. 1). Table 1 shows a summary of characteristics of these 17 studies. Summarised details of the included RCTs and observational studies, and of the identified previous systematic reviews, are included as supporting information; S3 Table: Summary details of included RCTs (n = 9), S4 Table: Summary details of included observational studies (n = 8), and S5 Table: Summary details of identified previous systematic reviews (n = 7).

**Table 1:** Characteristics of eligible studies (n = 17).

Study characteristics	Number of studies
**Study design**	
Randomised controlled trials [26]–[34]	9
Prospective cohort studies [6], [37], [38], [40], [41]	5
Other observational studies [35], [36], [39]	3
**Setting**	
Household or household-type transmission [6], [26], [27], [30], [33]–[36], [38]–[41]	12
Individual transmission [28], [29], [31], [32], [37]	5
**Mode of influenza infection**	
Natural means [6], [26]–[28], [30]–[41]	16
Artificial inoculation [29]	1
**Influenza type**	
Seasonal [6], [26]–[34]	10 (A(H3N2), A(H1N1), B)
Pandemic [35]–[41]	7 (A(H1N1)pdm09)
**Intervention (Neuraminidase inhibitor type)**	
Oseltamivir [6], [27]–[29], [34]–[38], [41]	10
Zanamivir [26], [30]–[33], [39]	6
Oseltamivir or zanamivir [40]	1

Specific study characteristics and the number of studies that possess each characteristic.

The RCTs (n = 9) [26]–[34] and observational studies (n = 8) [6], [35]–[41] involved 10,532 and 8,740 individuals respectively (total = 19,272). We found no articles on avian influenza that met our study eligibility criteria. All retrieved articles were either on oseltamivir, zanamivir or both. Of all studies, 12 (71%) evaluated transmission in households or discrete household-type settings [6], [26], [27], [30], [33]–[36], [38]–[41] and five (29%) evaluated individual transmission [28], [29], [31], [32], [37].

### Risk of bias within studies

**Figure 2:**
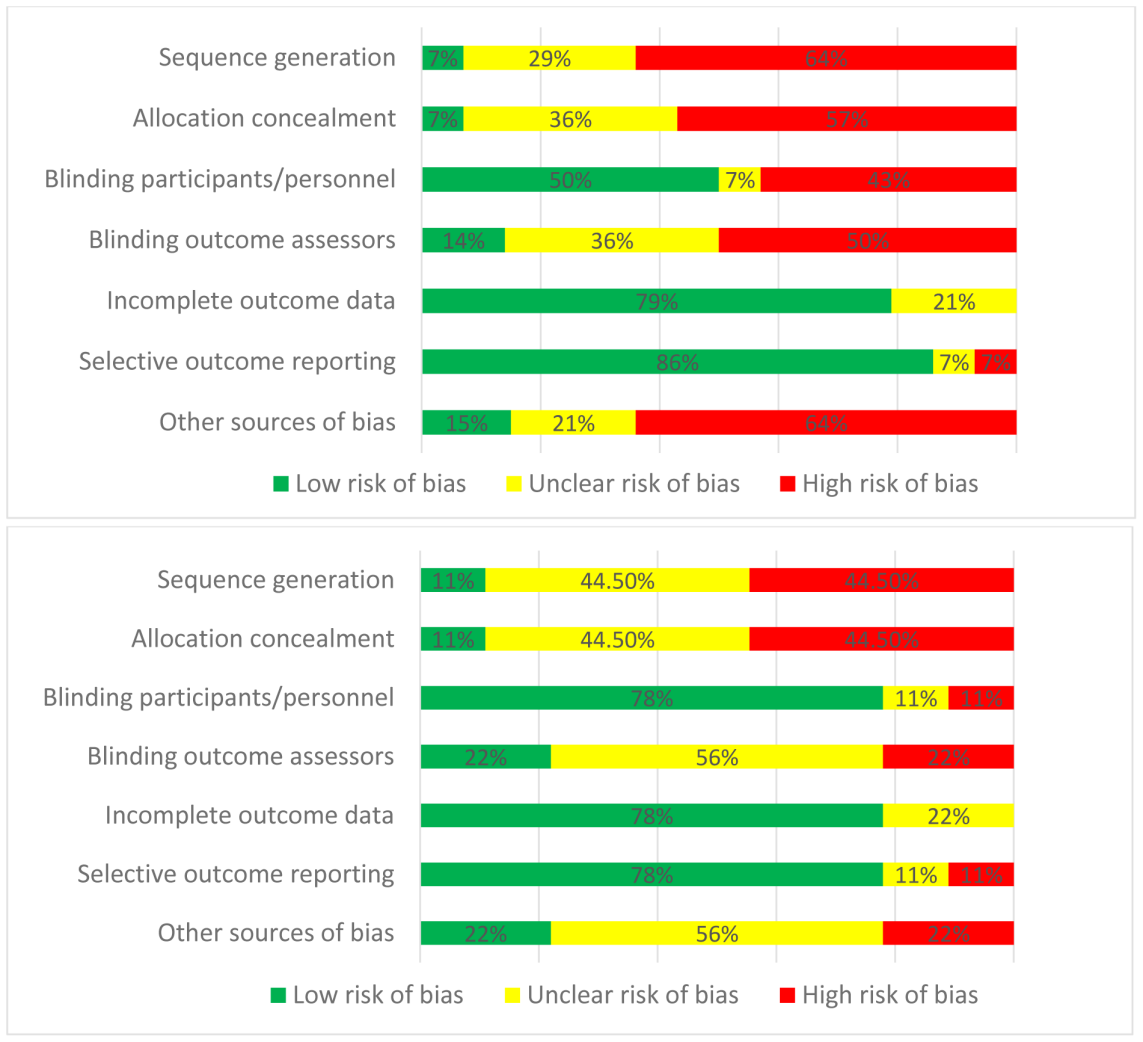
Risk of bias assessment using the Cochrane Collaboration tool. Upper panel: RCTs and prospective cohort studies (n = 14); Lower panel: RCTs only (n = 9).

Fig. 2 and Table 2 show the overall risk of bias per domain or question for the RCTs and observational studies. A high proportion of the RCTs was judged to be at high or unclear risk of bias for sequence generation, allocation concealment, blinding of outcome assessors and other sources of bias. However, most RCTs were at a low risk of bias for blinding of participants and personnel, incomplete outcome data and selective outcome reporting. The included prospective cohort studies were judged to be at high risk of bias using the Cochrane Collaboration tool while retrospective cohort studies were at low risk of bias within reporting domains of the Newcastle Ottawa scale. Furthermore, nearly all RCTs and observational studies presented additional risk of effect modification due to the vaccination status of participants (with inclusion of both vaccinated and unvaccinated participants). There were also variable proportions of comorbidities in each study sample population.

**Table 2:** Risk of bias assessment for observational studies (n = 3) excluding prospective cohort studies) using Newcastle Ottawa Quality Assessment Scale.

Study			
Domain	Goldstein et al. (2010)	Nishiura & Oshitani (2011)	Fallo et al. (2012)
Representativeness of the exposed cohort	**✓**	**✓**	**✓**
Selection of the non-exposed cohort	**✓**	**✓**	**✓**
Ascertainment of exposures	**✓**	**✓**	**✓**
Demonstration that outcome of interest was not present at start of study	**✓**	**✓**	**✓**
Comparability of cohorts on the basis of the design or analysis	**✗**	**✓**	**✗**
Assessment of outcome	**✗**	**✓**	**✗**
Was follow-up long enough for outcomes to occur	**✓**	**✓**	**✓**
Adequacy of follow-up of cohorts	**✓**	**✓**	**✓**

**✓** Denotes a score of 1 (domain assessment was satisfactory), **✗** denotes no score (domain assessment was not satisfactory).

### Synthesis of results

#### Prophylaxis with oseltamivir

**Figure 3:**
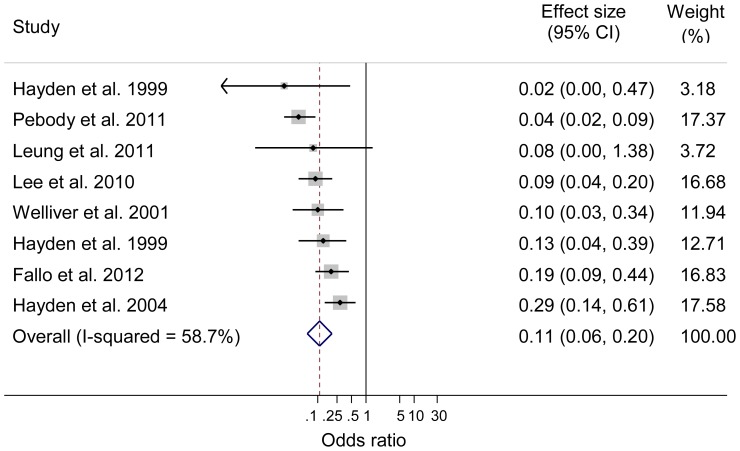
Meta-analysis of pre- and post-exposure prophylaxis with oseltamivir against seasonal and influenza A(H1N1)pdm09 (Individual protection). Horizontal axis represent odds ratio; Columns represent study authors and year of publication, effect size including pooled estimate of effect, and 95% CI, and the weighting of each study in the meta-analysis.

Four RCTs studied the use of oseltamivir for prophylaxis against laboratory confirmed seasonal influenza [27]–[29], [34]; three studied post-exposure prophylaxis [27], [29], [34], and one pre-exposure prophylaxis [28]. Oseltamivir was found to have 100% (p<0.001) [29], 68% (95% CI 34.9 to 84.2; p = 0.0017) [27], and 89% (95% CI 67 to 97; p<0.001) [34] protective efficacy for individuals against laboratory confirmed influenza when used post-exposure. For pre-exposure prophylaxis, protective efficacy was 87% (95% CI 65 to 96; p<0.001) [28]. Post-exposure prophylaxis against seasonal influenza provided statistically significant protective efficacy of 58.5% and 84% for household contacts respectively [27], [34]. Six observational studies evaluated oseltamivir for post-exposure prophylaxis against influenza A(H1N1)pdm09 [35]–[38], [40], [41] and one observational study against seasonal influenza [6]. The secondary attack rate (SAR) among household contacts was found to be lower in those given prophylaxis (0%) compared to those not (8.5%). There was a significant reduction in influenza R_0_ from 1.91 (95% CI 1.50 to 2.36) before prophylactic intervention to 0.11 after intervention (95% CI 0.05 to 0.20; Bayesian posterior hypothesis p<0.001) [37]. Meta-analysis of oseltamivir prophylaxis for individual protection irrespective of study design, influenza strain and combining pre- and post-exposure studies showed the pooled odds of laboratory confirmed influenza was statistically significantly lower compared to placebo or no therapy (n = 8 studies [27]–[29], [34], [35], [37], [38], [41]; OR = 0.11; 95% CI 0.06 to 0.20; p<0.001; I^2^ = 58.7%; see Fig. 3).

**Figure 4:**
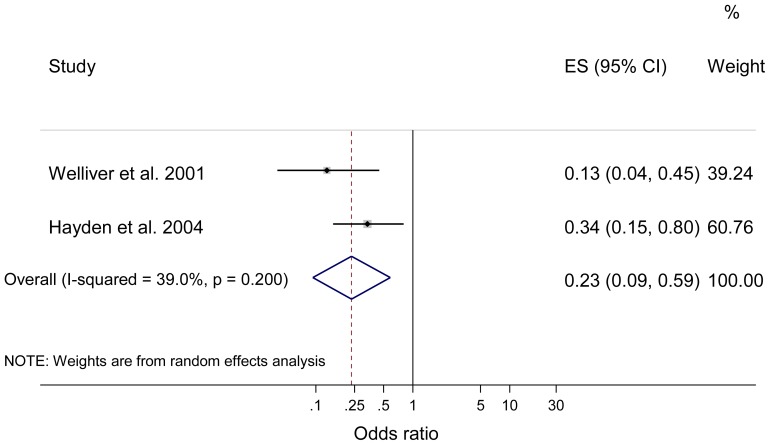
Meta-analysis of prophylaxis for households with oseltamivir against seasonal influenza. Horizontal axis represent odds ratio; Columns represent study authors and year of publication, effect size including pooled estimate of effect, and 95% CI, and the weighting of each study in the meta-analysis.

Sensitivity analyses of the oseltamivir data demonstrated pooled estimates for individual protection comparable to the primary analysis for experimental studies of seasonal influenza (n = 4 studies [27]–[29], [34]; OR = 0.15; 95% CI 0.07 to 0.33; p<0.001; I^2^ = 37.3%) and observational studies of influenza A(H1N1)pdm09 (n = 4 studies [35], [37], [38], [41]; OR = 0.09; 95% CI 0.04 to 0.19; p<0.001; I^2^ = 61.7%). Meta-analysis of prophylaxis with oseltamivir against seasonal influenza for household protection also showed statistically significantly lower pooled odds of laboratory confirmed influenza compared to placebo or no therapy (n = 2 studies [27], [34]; OR = 0.23; 95% CI 0.09 to 0.59; p<0.002; I^2^ = 39.0%; see Fig. 4).

#### Prophylaxis with zanamivir

**Figure 5:**
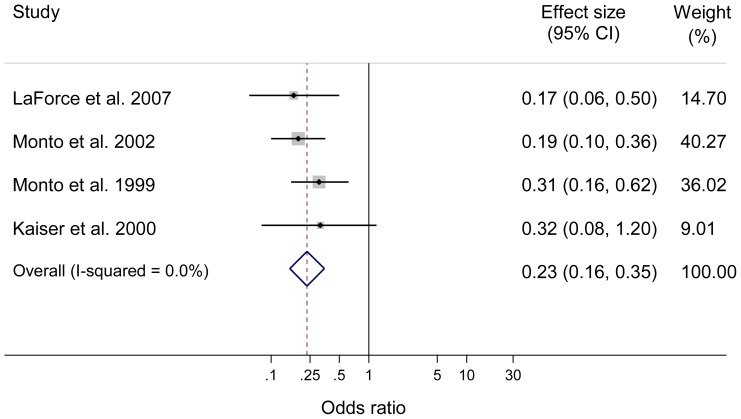
Meta-analysis of pre- and post-exposure prophylaxis with zanamivir against seasonal influenza (individual protection). Horizontal axis represent odds ratio; Columns represent study authors and year of publication, effect size including pooled estimate of effect, and 95% CI, and the weighting of each study in the meta-analysis.

**Figure 6:**
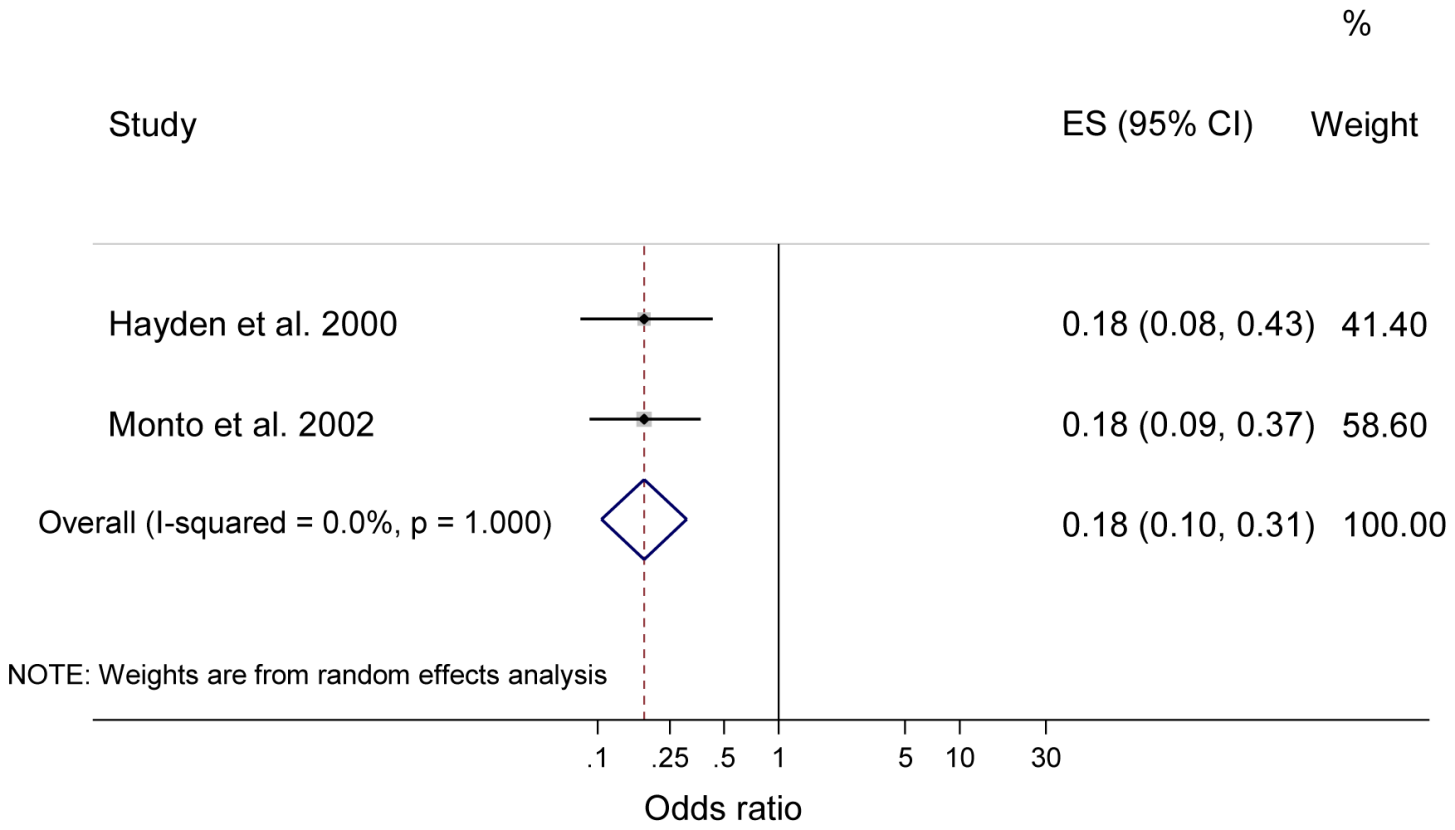
Meta-analysis of prophylaxis for households with zanamivir against seasonal influenza. Horizontal axis represent odds ratio; Columns represent study authors and year of publication, effect size including pooled estimate of effect, and 95% CI, and the weighting of each study in the meta-analysis.

There were five RCTs on zanamivir, all on seasonal influenza [26], [30]–[33]. Three of these reported data on individual protection [30]–[32], and one study each reported data on household protection [26], and both individual and household protection [33]. Zanamivir was found to have a protective efficacy of 83% and 84% for individuals against seasonal influenza with pre-exposure prophylaxis for 28 days (p<0.001) [31], [32]. Zanamivir was also found to have a protective efficacy of 82% (p<0.001) for individuals after 10 days post-exposure prophylaxis against seasonal influenza, and 73% protective efficacy (p = 0.058) after five days post-exposure prophylaxis [30], [33]. Zanamivir prophylaxis for 10 days was found to have a protective efficacy of 72% and 81% for households, with both results statistically significant (p<0.001). Meta-analysis of prophylaxis for individual protection with zanamivir (combined pre- and post-exposure prophylaxis) showed the pooled odds of laboratory confirmed influenza was statistically significantly lower compared to placebo or no therapy (n = 4 studies [30]–[33]; OR = 0.23; 95% CI 0.16 to 0.35; p<0.001; I^2^ = 0.0%; see Fig. 5). Similarly, meta-analysis of prophylaxis with zanamivir against seasonal influenza for household protection also showed the pooled odds of laboratory confirmed influenza was statistically significantly lower compared to placebo or no therapy (n = 2 studies [26], [33]; OR = 0.18; 95% CI = 0.10 to 0.31; p<0.001; I^2^ = 0.0%; see Fig. 6).

#### Treatment of index case alone with either oseltamivir or zanamivir

One observational study of 1547 households found that use of oseltamivir or zanamivir for treatment of an index case offered 43% (95% CI 27 to 56%) and 42% (95% CI 14 to 62%) household protection against secondary cases when the index case was treated within 24 hours or within 24 to 48 hours of symptom onset respectively (p value not given) [39].

#### Risk of bias across studies

We did not identify evidence of publication bias in any of the meta-analyses we carried out.

## Discussion

There have been controversies surrounding the evidence base on the effectiveness of neuraminidase inhibitors for influenza prevention and treatment. The recent Cochrane Collaboration review on published and unpublished data from only RCTs mainly in healthy individuals with mild illnesses due to seasonal influenza recommended a review of the guidance on using neuraminidase inhibitors based on the findings of small benefit of treatment compared with the risk of harm; [14] nevertheless the same review concludes that prophylactic use reduces the risk of developing symptomatic influenza. We felt it was important to include observational data, including that generated during the 2009–10 pandemic period which potentially inform clinical and public health practice. In addition we felt that the amalgamation of data on pre- and post-exposure use better suited the circumstances under which neuraminidase inhibitors might be used in a WHO Rapid Containment response setting. Our results suggest that zanamivir and oseltamivir are both effective as prophylaxis for individuals and households against laboratory confirmed seasonal influenza and influenza A(H1N1)pdm09 infection, irrespective of modality of usage (pre-exposure or post-exposure). We did not find any data reporting on the effectiveness of prophylaxis for a wider population group or for newer neuraminidase inhibitors. It is important to recognise that the WHO Rapid Containment Protocol anticipates that all (or almost all) asymptomatic residents of a population will be given neuraminidase inhibitor prophylaxis for 20 days. This will be an emergency measure, undertaken without drawing any distinction between those exposed (post-exposure prophylaxis) and unexposed (pre-exposure prophylaxis) within the containment zone; in effect a mixture of pre-exposure and post-exposure prophylaxis in unknown proportions. Therefore combining data on studies of pre- and post-exposure prophylaxis, as we have done in this review, offers the most meaningful estimate of effectiveness in the contest of the Rapid Containment Protocol and similar emergency public health control interventions in community settings. To our knowledge, it is the first systematic review to take such an approach.

Our data are broadly consistent with the findings from RCTs in the latest Cochrane Collaboration review on prophylaxis against symptomatic influenza [14], even though the two datasets are not fully overlapping in terms of RCTs included and the Cochrane review did not consider observational studies. Although we did not identify evidence of publication bias in any of the meta-analyses carried out, we cannot fully exclude this because there were relatively few studies available. It should also be noted that 37.5% of included oseltamivir studies and 50% of zanamivir studies respectively, emanated from essentially the same group of investigators for each drug; and all RCTs were sponsored by the respective manufacturers. Some caution is therefore needed during interpretation.

Overall our estimates of protection are highly consistent with those obtained by previous reviews. Cooper et al. 2003 reviewed two RCTs each on oseltamivir and zanamivir, and found a 70% to 90% reduction in odds of individuals developing laboratory confirmed influenza with oseltamivir or zanamivir as post-exposure prophylaxis. They showed a protective efficacy of 74% (95% CI 16 to 92%) for individuals with oseltamivir or zanamivir, 81% (95% CI 62% to 91%) with zanamivir for households and 90% (95% CI 71% to 96%) with oseltamivir for households. Langley and Faughnan 2004 reviewed six RCTs on oseltamivir and zanamivir, and found a reduced rate of laboratory confirmed influenza ranging from 18% to 67% in the placebo group to 3.6% to 38% in the chemoprophylaxis group. Jefferson et al. 2006 reviewed six RCTs and reported a 62% (95% CI 15% to 83%) protective efficacy with zanamivir for individuals, and 61% (95% CI 15% to 82%) and 73% (95% CI 33% to 89%) protective efficacy with 75 mg and 150 mg oseltamivir respectively (the latter being not the licensed dosage), for individuals against laboratory confirmed influenza. Jefferson et al. 2009 reviewed two RCTs each for oseltamivir and zanamivir, and found a protective efficacy of 62% (95% CI 15% to 83%) for individuals using zanamivir, and 61% (95% CI 15% to 82%) for oseltamivir when given as post-exposure prophylaxis against laboratory confirmed influenza. Shun-Shin et al. 2009 and Wang et al. 2012 reviewed three RCTs each and found an 8% absolute reduction in laboratory confirmed influenza with both zanamivir and oseltamivir prophylaxis. Jackson et al. 2011 reviewed two and three RCTs for oseltamivir and zanamivir respectively and showed a household protective efficacy of 81% (95% CI 55% to 92%) for oseltamivir, and 79% (95% CI 67% to 87%) for zanamivir against symptomatic laboratory confirmed influenza. All the RCTs included in the above previous systematic reviews form part of this present review; but individually, previous systematic reviews did not include all the RCTs. Therefore our review includes the largest number of RCTs.

In all previous systematic reviews, oseltamivir and zanamivir were compared against placebo using RCT designs. In our opinion, observational studies also form part of a comprehensive evaluation, especially since further evidence was generated during the 2009 pandemic when RCT designs were, in general, ethically unfeasible. Therefore inclusion of observational studies from large populations during pandemic periods, as we have done, may provide estimates that are more relevant for pandemic policy makers. It should be noted that the Rapid Containment Protocol is aimed at preventing influenza transmission in a large geographically cordoned population of typically over one million people. However, this contrasts sharply with the evidence base, which we found mainly restricted to household level studies. A smaller number of non-household studies, for example in military barracks and university community settings, also met our definition of community transmission but nevertheless these were still highly restricted examples, compared with the community transmission scenario envisaged in the Rapid Containment Protocol.

Studies included in this review varied in methodology including the participants selection criteria, influenza type, virus strain and virulence (in challenge studies), intervention type, dose and administration strategy, duration of intervention, comparators, study settings, and characteristics of study participants and rate of compliance. Variations in participants’ age distribution, gender, influenza vaccination status and comorbidities were judged to pose potential risks of heterogeneity within the same study types. In particular, we acknowledge that variations in participants’ influenza vaccination status could be an effect modifier in many of the studies, mostly observational, which lacked clarity on vaccination status of the study populations. In reality, under a rapid containment scenario, it is most likely that the population would be unvaccinated against the emerging virus. Information regarding sample size calculation was found to be lacking in many studies and among those providing such information, there were differences in the assumptions made for the calculation. Some studies were randomised by individual [28]–[32], while others were randomised by household [26], [27], [33], [34]. Variation in treatment compliance between studies is a potential limitation. While 98% compliance was reported in some studies [26]–[28], this information was not provided in many others. Compliance may also be an issue during any ‘real-life’ rapid containment operation.

A unique study evaluated the impact of ring chemoprophylaxis with oseltamivir on transmission of influenza A(H1N1)pdm09 and found a significant reduction in SAR from 6.4% before prophylaxis to 0.6% afterwards [37]. However, the study setting was a semi-closed military camp in Singapore, where strict adherence to directives and high compliance rate are both expected. Measures such as quarantine, treatment of infected individuals, and restriction of movement were also implemented. These would however be more difficult to actualise in a heterogeneous civilian population. Reduction of influenza spread is often given as a rationale for treatment of infected individuals. However, only one study on treatment of the index case alone met our criteria for inclusion. This study was observational, and evaluated household protection against laboratory confirmed influenza with the use of oseltamivir or zanamivir.

To adequately inform public health policy on influenza containment, it is necessary to evaluate all population-wide experimental and observational studies on the impact of neuraminidase inhibitors on seasonal, pandemic and avian influenza transmission, which this systematic review has sought to do. However, while it provides substantial evidence for oseltamivir and zanamivir effectiveness for individual and household prophylaxis, we did not identify direct evidence to confirm or refute the impact of neuraminidase inhibitors on community transmission in wider population settings.

## Conclusion

There is strong evidence that the neuraminidase inhibitors oseltamivir and zanamivir are effective as prophylaxis for individuals and households irrespective of modality of use (pre- and post-exposure) and treatment of the index case (or not). Beyond household settings, the evidence base is much more limited. We found no data which directly support an effect on community transmission as envisaged by the WHO Rapid Containment Protocol.

## Supporting Information

S1 Table **Literature search sources.** Cochrane Library = Cochrane Central Register of Controlled Trials; CDSR = Cochrane Database of Systematic Reviews; DARE = Database of Abstracts of Effects; NHS = National Health Services; HTA = Health Technology Assessment
(PDF)

S2 Table **Literature search terms.** MeSH = Medical Subject Headings
(PDF)

S3 Table **Summary details of included RCTs (n = 9).** PE = Protective Efficacy; RCT = Randomised Controlled Trial
(PDF)

S4 Table **Summary details of included observational studies (n = 8).** SAR = Secondary Attack Rate; OR = Odds Ratio; PE = Protective Efficacy
(PDF)

S5 Table **Summary details of identified previous systematic reviews (n = 7).** NAI = Neuraminidase Inhibitor; PE = Protective Efficacy; IR = Infection Rate; SAR = Secondary Attack Rate
(PDF)

S1 Checklist **PRISMA checklist.**
(DOC)
