# Supplementary material for: Use of Neuraminidase Inhibitors for Rapid Containment of Influenza: A Systematic Review and Meta-Analysis of Individual and Household Transmission Studies
Source: PLoS One. 2014 Dec 9;9(12):e113633. doi: 10.1371/journal.pone.0113633 (PMC4260958; doi:10.1371/journal.pone.0113633)
Supplement: S1 Table — Literature search sources. Cochrane Library = Cochrane Central Register of Controlled Trials; CDSR = Cochrane Database of Systematic Reviews; DARE = Database of Abstracts of Effects; NHS = National Health Services; HTA = Health Technology Assessment (PDF) [file pone.0113633.s001.pdf]

Table S1: Literature search sources

Categories	Sources
Healthcare databases	MEDLINE<br>EMBASE<br>CINAHL<br>Cochrane Library<br>PubMed<br>WHO Global Index Medicus
Evidence based reviews	Bandolier<br>Cochrane Library (CDSR, DARE, NHS HTA database)
Guidelines	NHS Evidence (NHS Clinical Knowledge Summaries and the National Library of Guidelines)
Grey Literature	Web of Science<br>NHS Evidence (drug information, evidence summaries, grey literature, health technology assessments, primary research, and systematic reviews) OpenSIGLE (System for information on grey literature in Europe)<br>Neuraminidase inhibitors manufacturers (Roche, GlaxoSmithkline, Biota Scientific)<br>IFPMA (International Federation of Pharmaceutical Manufacturers Associations, Geneva/Zurich)
Hand searching of relevant journals	Journal of Infectious Diseases, Clinical Infectious Diseases Journal, British Medical Journal
Internet searching	www.google.com<br>www.who.int<br>www.cdc.gov<br>www.flu.gov<br>www.dh.gov.uk<br>www.hpa.org.uk
Referencing tracking	Reference list of all selected studies will be searched to identify further relevant studies
Citation tracking	Web of Science (Science Citation Index)<br>Google Scholar
