# Supplementary material for: Use of Neuraminidase Inhibitors for Rapid Containment of Influenza: A Systematic Review and Meta-Analysis of Individual and Household Transmission Studies
Source: PLoS One. 2014 Dec 9;9(12):e113633. doi: 10.1371/journal.pone.0113633 (PMC4260958; doi:10.1371/journal.pone.0113633)
Supplement: S2 Table — Literature search terms. MeSH = Medical Subject Headings (PDF) [file pone.0113633.s002.pdf]

Table S2: Literature search terms

Area	MeSH thesaurus headings	Free text
Population	persons	patient* OR health* OR neonat* OR infant OR child* OR young OR adolescen* OR adult* OR elder* OR old* OR men OR women OR people OR person* OR subject* OR individual*
	disease	disease* OR ill* OR risk
	pediatrics	paediatr* OR pediatr*
	geriatrics	geriatr*
	polymerase chain reaction	rt-pcr OR rtpcr OR (reverse AND transcriptase AND polymerase AND chain AND reaction) OR (polymerase AND chain AND reaction)
		laboratory AND culture
		laboratory AND confirm*
Intervention	oseltamivir	oseltamivir OR tamiflu
	zanamivir	zanamivir OR relenza
		laninamivir OR inavir
	antiviral agents	influenza-antiviral* OR flu-antiviral* OR (influenza AND antiviral*) OR (flu AND antiviral)
		neuraminidase AND inhibitor*
		neuraminidase OR antiviral* AND (drug* OR therap* OR treat*)
Comparators	placebos	(no OR placebo* OR sham) AND (oseltamivir OR zanamivir OR laninamivir OR tamiflu OR relenza OR inavir OR influenza-antiviral* OR flu-antiviral* OR (influenza AND antiviral*) OR (neuraminidase AND inhibitor*) OR (neuraminidase OR antiviral*) AND (drug* OR therap* OR treat*))
Outcomes	disease transmission, infectious	transmi* OR spread OR infect* OR "person to person" OR "person-person" OR "person-to-person" OR house* OR communit*

Influenza terms	influenza, human	influenza OR flu OR (influenza AND like) OR influenza-like OR (flu AND like) OR flu-like OR ILI
	influenza A virus	
	influenza B virus	
