# Supplementary material for: Use of Neuraminidase Inhibitors for Rapid Containment of Influenza: A Systematic Review and Meta-Analysis of Individual and Household Transmission Studies
Source: PLoS One. 2014 Dec 9;9(12):e113633. doi: 10.1371/journal.pone.0113633 (PMC4260958; doi:10.1371/journal.pone.0113633)
Supplement: S3 Table — Summary details of included RCTs (n = 9). PE = Protective Efficacy; RCT = Randomised Controlled Trial (PDF) [file pone.0113633.s003.pdf]

Table S3: Summary details of included RCTs (n = 9)

Citation & Country	Study type	Influenza type & transmission	Intervention	Duration of intervention	Comparator	Outcome measure	Results
**Hayden et al. 1999**<br><br>USA	RCT	Seasonal (Individual)<br><br>A(H1N1)	Oseltamivir 100mg oral	5 days	Placebo	PE	100% PE for individual against laboratory confirmed influenza (p<0.001)<br><br>61% PE for individual against serology confirmed influenza (p=0.16)
**Hayden et al. 1999**<br><br>USA	RCT	Seasonal (Individual)<br><br>A(H1N1), A(H3N2) & B	Oseltamivir 75mg oral	42 days	Placebo	PE	87% (95% CI 65-96%) PE for individual against laboratory confirmed influenza (p<0.001)<br><br>74% (95% CI 53-88%) PE for individual against influenza-like illness (p<0.001)
**Monto et al. 1999**<br><br>USA	RCT	Seasonal (Individual)<br><br>A(H3N2)	Zanamivir 10mg inhaled	28 days	Placebo	PE	67% (95% CI 39-83%) PE for individual against laboratory confirmed influenza (p<0.001)<br><br>84% (95% CI 55-94%) PE for individual against laboratory confirmed influenza with fever (p<0.001)
**Kaiser et al. 2000**<br><br>North America & Europe	RCT	Seasonal (Household-type)<br><br>A(H3N2)	Zanamivir 10 mg inhaled & 16mg/ml intranasal sprays	5 days	Placebo	PE	73% PE for individual against laboratory confirmed influenza (p=0.058)
**Hayden et al. 2000**<br><br>USA, Canada, UK & Finland	RCT	Seasonal (Household) A(H3N2) & B	Zanamivir 10mg inhaled	10 days	Placebo	PE	72% (95% CI 42-87%) PE for household against laboratory confirmed influenza (p<0.001)

**Welliver et al. 2001**<br><br>North America & Europe	RCT	Seasonal (Household)<br><br>A(H3N2) & B	Oseltamivir 75mg oral	7 days	Placebo	PE	89% (95% CI 67-97%) PE for individual against laboratory confirmed influenza (p<0.001)<br><br>84% (95% CI 49-95%) PE for household against laboratory confirmed influenza (p<0.001)
**Monto et al. 2002**<br><br>North America, Europe, Australia, New Zealand & S/Africa	RCT	Seasonal (Household)<br><br>A(H1N1), A(H3N2) & B	Zanamivir 10mg inhaled	10 days	Placebo	PE	81% (95% CI 64-90%) PE for household against laboratory confirmed influenza (p<0.001)<br><br>82% PE for individual against laboratory confirmed influenza (p value not provided)
**Hayden et al. 2004**<br><br>North America & Europe	RCT	Seasonal (Household)<br><br>A(H1N1) & B	Oseltamivir 75mg oral	10 days (prophylaxis)<br><br>5days (treatment of case)	Treatment alone	PE	58.5% (95% CI 15.6-79.6%) PE for household against laboratory confirmed influenza (p =0.0114)<br><br>68% (95% CI 34.9-84.2%) PE for individual against laboratory confirmed influenza (p = 0.0017)
**LaForce et al. 2007**<br><br>USA, Canada Czech Republic France, Latvia & Germany	RCT	Seasonal (Individual)<br><br>A(H1N1), A(H3N2) & B	Zanamivir 10mg inhaled	28 days	Placebo	PE	83% PE for individual against laboratory confirmed influenza (p<0.001)
