# Supplementary material for: Use of Neuraminidase Inhibitors for Rapid Containment of Influenza: A Systematic Review and Meta-Analysis of Individual and Household Transmission Studies
Source: PLoS One. 2014 Dec 9;9(12):e113633. doi: 10.1371/journal.pone.0113633 (PMC4260958; doi:10.1371/journal.pone.0113633)
Supplement: S4 Table — Summary details of included observational studies (n = 8). SAR = Secondary Attack Rate; OR = Odds Ratio; PE = Protective Efficacy (PDF) [file pone.0113633.s004.pdf]

Table S4: Summary details of included observational studies (n = 8)

Citation & Country	Study type	Influenza type & transmission	Intervention	Duration of intervention	Comparator	Outcome measure	Results
**Odaira et al. 2009**<br><br>Japan	Observational	Pandemic (Household)<br><br>A(H1N1)pdm09	Oseltamivir 75mg oral<br><br>Zanamivir 10mg inhaled	7 – 10 days	No intervention	SAR	SAR of 7.6% in no intervention household against 0.8% in intervention household (p values not provided, however result was stated to be non-significant)
**Ng et al. 2010**<br><br>Hong Kong	Observational	Seasonal (Household)<br><br>A(H1N1), A(H3N2) & B	Oseltamivir (dose not clear)	7 – 10 days	No antiviral	Adjusted OR	Lower OR: 0.54 (95% CI 0.11-2.57) of laboratory confirmed influenza in individual when intervention was commenced within 24 hours of symptom onset compared to no intervention
**Goldstein et al. 2010**<br><br>USA	Observational	Pandemic (Household) A(H1N1)pdm09	Oseltamivir (dose not clear)	Not clear	No antiviral	PE	42% PE (OR: 0.58; 95% CI 0.19-1.73) for household and 50% PE (OR: 0.5; 95% CI 0.17-1.46) for individual (intervention within 24 hours of symptom onset compared to within 72 hours or no intervention; p values not provided)
**Lee et al. 2010**<br><br>Singapore	Observational	Pandemic (Individual)<br><br>A(H1N1)pdm09	Oseltamivir 75mg oral	10 days	No antiviral	SAR (Reproductive rate (R_0))	SAR of 6.4% (R_0=1.91[95% CI 1.50-2.36]) in community without intervention<br><br>SAR of 0.6% (R_0=0.11 [95% CI 0.05-0.20]) in community after intervention (posterior hypothesis p<0.001 for R_0 result)
**Leung, Li & Chuang 2011**<br><br>Hong Kong	Observational	Pandemic (Household)<br><br>A(H1N1)pdm09	Oseltamivir (dose not clear)	10 days	No intervention	SAR	SAR of 8.5% in household individuals given no intervention compared to SAR of 0% in household individuals given oseltamivir prophylaxis (p value not provided)
**Pebody et al. 2011**<br><br>UK	Observational	Pandemic (Household)<br><br>A(H1N1)pdm09	Oseltamivir (dose not clear)	10 days	No intervention	SAR	SAR of 31.5% (95% CI 24.4-39.5%) among household members without intervention<br>SAR of 1.8% (95% CI 0.8-3.9%) among household members with intervention<br>SAR significantly higher in <16 years than >50 years (18.9 vs. 1.2%; p<0.001)

**Nishiura and Oshitani 2011**<br><br>Japan	Observational	Pandemic (Household)<br><br>A(H1N1)pdm09	Oseltamivir<br>&<br>Zanamivir (doses not clear)	Not clear	No treatment<br>&<br>Treatment commencement time	PE	43% (95% CI 27-56%) and 42% (95% CI 14-62%) PE for household when index case is treated within 24 hours and 24-48 hours respectively, compared to after 48 hours or no treatment (p value not provided)
**Fallo et al. 2012**<br><br>Argentina	Observational	Pandemic (Household)<br><br>A(H1N1)pdm09	Oseltamivir (dose not clear)	Not clear	No antivirals	SAR	SAR of 38.7% in household individuals without intervention compared with<br>SAR of 10.9% in household individuals with intervention<br>(p value not provided)
