# Supplementary material for: Use of Neuraminidase Inhibitors for Rapid Containment of Influenza: A Systematic Review and Meta-Analysis of Individual and Household Transmission Studies
Source: PLoS One. 2014 Dec 9;9(12):e113633. doi: 10.1371/journal.pone.0113633 (PMC4260958; doi:10.1371/journal.pone.0113633)
Supplement: S5 Table — Summary details of identified previous systematic reviews (n = 7). NAI = Neuraminidase Inhibitor; PE = Protective Efficacy; IR = Infection Rate; SAR = Secondary Attack Rate (PDF) [file pone.0113633.s005.pdf]

Table S5: Summary details of identified previous systematic reviews (n = 7)

Citation	Study type	Influenza type	NAI	Duration of intervention	Comparator	Outcome measure	Results
**Cooper et al. (2003)**	Systematic review & Meta-analysis	Seasonal A(H3N2), A(H1N1) B	Oseltamivir (based on 2 RCTs) & Zanamivir (based on 2 RCTs)	varied	Placebo	PE	70 – 90% reduction in odds of developing laboratory confirmed influenza (with either oseltamivir or zanamivir)<br><br>Zanamivir 81% (95% CI 62-91%) protection for households<br>Oseltamivir 74% (95% CI 16-92%) for individuals<br>90% (95% CI 71-96%) for households
**Langley & Faughnan (2004)**	Systematic review	Seasonal A(H3N2), A(H1N1) B	Oseltamivir & Zanamivir (based on 6 RCTs)	varied	Placebo	IR	18-67% in the placebo group<br>3.6-38% in prophylaxis group
**Jefferson et al. (2006)**	Systematic review	Seasonal A(H3N2), A(H1N1) B	Oseltamivir (based on 3 RCTs) & Zanamivir (based on 3 RCTs)	varied	Placebo	PE	Protection for individual<br><br>10mg Zanamivir 62% (95% CI 15-83%)<br>75mg Oseltamivir 61% (95% CI 15-82%)<br>150mg oseltamivir 73% (95% CI 33-89%)
**Jefferson et al. (2009)**	Systematic review & Meta-analysis	Seasonal A(H3N2), A(H1N1) B	Oseltamivir (based on 2 RCTs) & Zanamivir (based on 2RCTs)	varied	Placebo	PE	Prophylaxis with Zanamivir 62% (95% CI 15-83%)<br><br>With oseltamivir 61% (95% CI 15-82%)<br>With specifically 150mg oseltamivir daily 73% (95% CI 33-89%) against lab confirmed influenza
**Shun-Shin et al. (2009)**	Systematic review & Meta-analysis	Seasonal A(H3N2), A(H1N1) B	Oseltamivir (based on 1RCT) & Zanamivir (based on 2 RCTs)	varied	Placebo	SAR	8% (95% CI 5-12%) decrease in the incidence of influenza with 10 days post-exposure prophylaxis with oseltamivir or zanamivir
**Jackson et al. (2011)**	Systematic review	Seasonal A(H3N2), A(H1N1) B	Oseltamivir (2 RCTs) & Zanamivir (3 RCTs)	varied	Placebo	PE	Prophylaxis for households against symptomatic laboratory confirmed influenza<br>Oseltamivir 81% (95% CI 55-92%)<br>Zanamivir 79% (95% CI 67-87%)

**Wang et al. (2012)**	Systematic review	Seasonal<br><br>A(H3N2),<br>A(H1N1)<br>B	Oseltamivir<br>(based on 1 RCT)<br>&<br>Zanamivir<br>(based on 2 RCTs)	varied	Placebo	IR	8% absolute reduction in influenza rate with either zanamivir or oseltamivir ($p<0.001$)
